# The HigB/HigA toxin/antitoxin system of *Pseudomonas aeruginosa* influences the virulence factors pyochelin, pyocyanin, and biofilm formation

**DOI:** 10.1002/mbo3.346

**Published:** 2016-03-14

**Authors:** Thammajun L. Wood, Thomas K. Wood

**Affiliations:** ^1^ Department of Chemical EngineeringPennsylvania State UniversityUniversity ParkPennsylvania16802; ^2^ Huck Institutes of the Life SciencesPennsylvania State UniversityUniversity ParkPennsylvania16802; ^3^ Department of Biochemistry and Molecular BiologyPennsylvania State UniversityUniversity ParkPennsylvania16802

**Keywords:** biofilm, HigA, HigB, pyochelin, pyocyanin, toxin/antitoxin system, virulence factors

## Abstract

Toxin/antitoxin (TA) systems are prevalent in most bacterial and archaeal genomes, and one of the emerging physiological roles of TA systems is to help regulate pathogenicity. Although TA systems have been studied in several model organisms, few studies have investigated the role of TA systems in pseudomonads. Here, we demonstrate that the previously uncharacterized proteins HigB (unannotated) and HigA (PA4674) of *Pseudomonas aeruginosa* PA14 form a type II TA system in which antitoxin HigA masks the RNase activity of toxin HigB through direct binding. Furthermore, toxin HigB reduces production of the virulence factors pyochelin, pyocyanin, swarming, and biofilm formation; hence, this system affects the pathogencity of this strain in a manner that has not been demonstrated previously for TA systems.

## Introduction

*Pseudomonas aeruginosa* is an opportunistic, Gram‐negative pathogen (Mace et al. 2008), and it is the primary cause of important chronic infections including those associated with cystic fibrosis (CF) (Moker et al. 2010), burn wound infections, bacterial keratitis, and urinary and peritoneal dialysis catheter infections (Mace et al. 2008). It is difficult to treat infections from *P. aeruginosa* because this species is highly tolerant to antibiotics (Lewis 2010). For example, late isolates of *P. aeruginosa* from CF patients can be high‐persister mutants with 100‐fold greater antibiotic tolerance (Mulcahy et al. 2010). The cause of antibiotic tolerance in many strains is thought to be due to activation of toxins of toxin/antitoxin (TA) systems (Jayaraman 2008; Lewis 2008) which causes dormancy by inactivating key metabolic functions like protein and ATP production (Kwan et al. 2013; Wood et al. 2013).

The first TA operons were discovered over 30 years ago (Ogura and Hiraga 1983) for stabilizing low copy number plasmids via postsegregational killing (Gerdes et al. 1986) and some additional roles of TA systems in cell physiology have become clear. For example, they are antiphage measures (Pecota and Wood 1996; Hazan and Engelberg‐Kulka 2004; Fineran et al. 2009), and TA systems slow metabolism to allow cells to withstand stress such as that from antibiotics (Cheng et al. 2014; Islam et al. 2015) and bile acid (Kwan et al. 2015). Furthermore, the expression of the toxin genes are induced under stress conditions (Aizenman et al. 1996; Sat et al. 2001; Hazan et al. 2004), and antitoxins like MqsA and DinJ directly control the stress response by regulating the stress response sigma factor RpoS (Wang et al. 2011; Hu et al. 2012). TA systems also have a role in biofilm formation (Ren et al. 2004; González Barrios et al. 2006; Kim et al. 2009).

Beyond phage inhibition, stress response, and biofilm formation, the role of TA systems in pathogenicity is also becoming more clear. Production of almost any active toxin to the point of making the cell dormant increases antibiotic tolerance (i.e., persistence) (Wang and Wood 2011), and deletion of some single TA systems decreases persistence (Dörr et al. 2010; Kim and Wood 2010). Although *Mycobacterium tuberculosis* has about 88 putative TA systems (Ramage et al. 2009), the nonpathogenic *Mycobacterium smegmatis* has only two putative TA systems, so pathogenicity might be related to the number of TA systems (Yamaguchi and Inouye 2011). As anticipated, inactivation of three of *M. tuberculosis* MazF/MazE TA systems reduced its persistence in vitro, its survival in macrophages, and its cell numbers in the spleen and lungs of guinea pigs (Tiwari et al. 2015). Similarly, TA systems play a role in the persistence of *Salmonella typhimurium* in macrophages in a mouse model for typhoid fever (Helaine et al. 2014). There are at least 11 type II TA systems in *S*. *typhimurium*, and several of them are conserved in other pathogenic bacteria but absent from other nonpathogenic strains (De la Cruz et al. 2013). Also, inactivation of three Vap‐type TA systems reduced virulence for nontypeable *Haemophilus influenzae* (NTHi) in a chinchilla model for otitis media (Ren et al. 2012), and inactivation of YoeB/YefM, Hha/TomB (García Contreras et al. 2008), and PasT/PasI TA systems are important for uropathogenic *Escherichia coli* infections in the bladder and spleen in murine models (Norton and Mulvey 2012). Hence, determining the function of TA systems and the target of these toxins is very important because of their roles in bacterial physiology and pathogenicity (Yamaguchi and Inouye 2011).

One prominent TA system found in many pathogens is HigB/HigA in which the antitoxin HigA masks the toxicity of the toxin HigB. Genes for the HigB/HigA TA system are found on the Rts1 plasmid originally isolated from *Proteus vulgaris* and are found in the chromosomes of the pathogens *Vibrio cholera* (Christensen‐Dalsgaard and Gerdes 2006; Budde et al. 2007), *Streptococcus pneumonia* (Chan et al. 2012), *Acinetobactor baumanni* (Jurenaite et al. 2013), *S. typhimurium* (De la Cruz et al. 2013), *Yersinia pestis* (Goulard et al. 2010), *M. tuberculosis* (Schuessler et al. 2013), *E. coli* CFT073 (Pandey and Gerdes 2005), and *E. coli* O157:H7 (Pandey and Gerdes 2005) and is also present in *E. coli* K12 (Christensen‐Dalsgaard et al. 2010). Additionally, *higBA* is widespread in *P. aeruginosa* clinical isolates (Williams et al. 2011). The HigB/HigA system has the uncommon gene arrangement with the toxin gene (*higB*) upstream of the antitoxin gene (*higA*), which is the reverse arrangement of most other TA systems (Tian et al. 1996).

HigB functions as an endoribonuclease in *Proteus* spp. (Hurley and Woychik 2009), *V. cholera* (Christensen‐Dalsgaard and Gerdes 2006), *A. baumanni* (Jurenaite et al. 2013), and *E. coli* K12 (Christensen‐Dalsgaard et al. 2010). Although the mechanism of how the SehA (*S* *almonella* *e* *nterica* Hig‐like) toxin works is unknown, it shares 40% identity with HigB from *E. coli* K12 (De la Cruz et al. 2013); hence, SehA is likely to be an endoribonuclease and have the same target as HigB (De la Cruz et al. 2013). SehAB was also found to play an important role in virulence in mice (De la Cruz et al. 2013).

Here, we identified and characterized the HigB/HigA system in the chromosome of *P. aeruginosa* PA14. The HigB/HigA system is shown to be a bona fide TA system. Moreover, the HigB/HigA system affects the virulence factors of the strain in a fashion that has not been demonstrated previously for TA systems, since activation of toxin HigB reduces pyocyanin, a toxin produced and secreted by *P. aeruginosa*, reduces the siderophore pyochelin, reduces swarming, and reduces biofilm formation. The closest HigB homolog to that we identify here in *P. aeruginosa* has only 34.3% identity (from *V. cholera*), so our findings are for a novel HigB/HigA TA system.

## Experimental Procedures

### Bacterial strains and culture conditions

The strains and plasmids used in this study are shown in Table 1. All strains were grown in lysogeny broth (LB) (Sambrook and Russell 2001) at 37°C except for the biofilm assay where M9 medium with 0.4% glucose and 0.4% casamino acids (Rodriguez and Tait 1983) was used. Chloramphenicol (30 *μ*g/mL) was used to maintain pCA24N‐based plasmids (Kitagawa et al. 2005) in *E. coli*. To obtain the specific growth rates, the *P. aeruginosa* PA14 wild‐type and *higA‐*mutant strains were inoculated into LB medium at an initial turbidity of 0.05 at 600 nm, and the turbidity at 600 nm was measured every an hour. At least two replicates were performed.

**Table 1:** Bacterial strains and plasmids used in this study

Strains or plasmids	Description	Source
Strains		
*Escherichia coli* TG1	*supE thi‐1 Δ(lac‐proAB) Δ(mcrB‐hsdSM)5*,* (r*_*K*_^*−*^ *m*_*K*_^*−*^ *)* F′ [*traD36 proAB* ^*+*^ *lacI* ^*q*^ *lacZΔM15*]	Sambrook et al. (1989)
*Pseudomonas aeruginosa* PA14	Wild type	Liberati et al. (2006)
*P. aeruginosa higA*	Gm^R^, *MAR2xT7* transposon insertion	Liberati et al. (2006)
Plasmids		
pCA24N	Cm^R^; *lacI* ^q^	Kitagawa et al. (2005)
pCA24N‐lacZ	Cm^R^; *lacI* ^q^ P_*T5‐lac*_::*His*_*6*_ *‐lacZ* ^+^	Kitagawa et al. (2005)
pCA24N‐His‐higB	Cm^R^; *lacI* ^q^ P_*T5‐lac*_::*His*_*6*_ *‐higB* ^+^	This study
pCA24N‐ higA‐FLAG	Cm^R^; *lacI* ^q^ P_*T5‐lac*_::*His*_*6*_ *‐higA‐flag* ^+^	This study
pCA24N‐ His‐higB‐higA‐flag	Cm^R^; *lacI* ^q^ P_*T5‐lac*_:: *His*_*6*_ *‐higB* ^+^, *higA‐flag* ^+^	This study
pCA24N‐ His‐higB‐higAX‐FLAG	Cm^R^; *lacI* ^q^ P_*T5‐lac*_:: *His*_*6*_ *‐higB* ^+^, *higA‐flag* ^+^ with ATG (start codon) mutated to ACC and with ATG (16–18 nt relative to start codon) mutated to ACCG	This study

Gm^R^ and Cm^R^ denote gentamycin and chloramphenicol resistance, respectively.

### Bioinformatics search

The web‐based search tool RASTA (Sevin and Barloy‐Hubler 2007) was used to search for type II TA systems. The search was performed against the complete genome sequence of *P. aeruginosa* PA14 (Lee et al. 2006) with standard parameters.

### Plasmid construction

Plasmid pCA24N(His‐higB) was constructed by amplifying the *higB* gene from the chromosome of *P. aeruginosa* PA14 (Lee et al. 2006) and cloning into pCA24N at the *Not*I and *Pst*I restriction sites placed *higB* under the control of *T5‐lac* promoter. The *higB* gene was fused with His tag in the pCA24N plasmid at the N terminus. Plasmids pCA24N(higA‐FLAG) and pCA24N(His‐higB‐higA‐FLAG), Fig. 3B, were constructed by amplifying the *higA* gene from the chromosome of *P. aeruginosa* PA14 with the addition of a FLAG tag at the C terminus and cloning into pCA24N and pCA24N(His‐higB), respectively, at the *Pst*I and *Hind*III restriction sites. All plasmids were verified by DNA sequencing. All oligonucleotides were synthesized by Integrated DNA Technologies (Coralville, IA), and the primer sequences are listed in Table 2.

**Table 2:** Oligonucleotides used for cloning and sequencing

Primer name	Sequence (5′→3′)
Cloning and sequencing	
HigB‐NotI‐f	TTTTTTGCGGCCGCTAATTCTGACCTTTCG
HigB‐PstI‐r	TTTTTTCTGCAGTCAGTGGTAATCAACTATTTCGAC
HigA‐PstI‐f	TTTTTTTTCTGCAGATTAAAGAGGAGAAATTAACTATGAGGAGGTGGACCATGGC
HigA‐FLAG‐HindIII‐r	TTTTTGTCGACAAGCTTCTACTTGTCATCGTCGTCCTTGTAGTCCTTGTCATCGTCGTCCTTGTAGTCTCCGTGAGCAAGCAGCGGCTCA
HigA‐PstI‐start‐QC‐f	ACCACTGACTGCAGATTAAAGAGGAGAAATTAACTACCAGGAGGTGGACCACCGCTACCA
pCA24N‐f	GCCCTTTCGTCTTCACCTCG
pCA24N‐r	GAACAAATCCAGATGGAGTTCTGAGGTCATT
Northern blot	
* ompA*‐f	CACTGGCTGGTTTCGCTACCG
* ompA*‐r	ACCCATTTCAAAGCCAACATC
* ompF*‐f	AAGCGCAATATTCTGGCAGT
* ompF*‐r	TGCCACCGTAACTGTTTTCA

All restriction enzyme sites are underlined. f indicates forward primer and r indicates reverse primer.

### Site‐directed mutagenesis of *higA*

Plasmid pCA24N(His‐higB‐higA‐FLAG) was used as a PCR template to replace the start codon of HigA with Thr; since methionine residues exist at amino acid positions 1 and 6, both methionines were converted to threonine (i.e., M1T and M6T) in case either one is the start codon. The primers higA‐PstI‐start‐QC‐f and higA‐FLAG‐HindIII‐r (Table 2) were used in the PCR reaction to generate the mutations. The PCR product was cloned into pCA24N(His‐higB) using the *Pst*I and *Hind*III sites. The resulting plasmid pCA24N(His‐higB‐higAX‐FLAG) was sequenced to confirm the mutations.

### Random mutagenesis of *higB* toxin

The plasmid pCA24N(His‐higB) was used for the error‐prone PCR template. Using the pCA24N‐f and pCA24N‐r primers (Table 2), *higB* was randomly mutated under error‐prone conditions (0.5 mmol/L Mn^2+^ and 5 mmol/L Mg^2+^) (Cadwell and Joyce 1992). Mutated *higB* inserts were digested and cloned into pCA24N at the *Not*I and *Pst*I restriction sites. Ligated product was electroporated into *E. coli* TG1. The electroporated population was plated on LB–chloramphenicol agars. To select for growth, recovered colonies were restreaked on LB–chloramphenicol agar with 1 mmol/L IPTG. Variants were chosen for DNA sequencing analysis and retransformation tests.

### Toxicity assay

Overnight cultures of strains *E. coli* TG1/pCA24N, *E. coli* TG1/pCA24N(lacZ), *E. coli* TG1/pCA24N(His‐higB), *E. coli* TG1/pCA24N(higA‐FLAG), *E. coli* TG1/pCA24N(His‐higB‐higA‐FLAG), and *E. coli* TG1/pCA24N(His‐higB‐higAX‐FLAG) were inoculated into 25 mL of LB medium at an initial of turbidity of 0.05 at 600 nm. IPTG (0.01 mmol/L) was added after 1 h, and the turbidity was recorded to determine growth.

### Western blot analysis and pull‐down assay

*Escherichia coli* TG1/pCA24N(His‐higB) and *E. coli* TG1/pCA24N(His‐higB‐higA‐FLAG) were inoculated into LB medium from the overnight culture at an initial turbidity of 0.05 at 600 nm, then 0.1 mmol/L of IPTG was added to produce HigB and HigA for 5 h. The cell pellets were resuspended in buffer (50 mmol/L NaCl and 20 mmol/L Tris, pH 7.4) with protease inhibitor, and the cells were lysed using a French Press (Thermo Electron, Waltham, MA); centrifugation (15,000*g* for 1 h at 4°C) was used to remove cell debris. The lysate was filtered through a 0.22‐*μ*m membrane and loaded on a HisTrap HP column (GE Healthcare, Pittsburgh, PA, USA). HigB and associated proteins were eluted with a 5–500 mmol/L imidazole gradient. The fractions containing HigB and HigA were identified using western blot analysis using HRP‐conjugated anti‐His antibody and HRP‐conjugated anti‐FLAG antibody (Thermo Scientific, Waltham, MA, USA). Blotted proteins were detected using the chemiluminescence reagents from the SuperSignal West‐Pico Chemiluminescence kit (Thermo Scientific).

### Northern blot analysis

*Escherichia coli* TG1/pCA24N(His‐higB) was inoculated from the overnight culture at an initial turbidity of 0.05 at 600 nm, then 0.5 mmol/L of IPTG was added to produce HigB for 3 h. The samples were collected at 0, 1 15, 30, and 60 min after induction. Total RNA was isolated using RNeasy Mini kit (Qiagen, Hilden, Germany). The *ompA* (outer membrane porin protein A) and *ompF* (outer membrane porin protein F) (Hurley and Woychik 2009) DNA probes were amplified from the chromosomal DNA of *E. coli* TG1/pCA24N(His‐higB) using primers *ompA*‐f and *ompA*‐r and *ompF*‐f and *ompF*‐r, respectively. The probes were labeled using Biotin 3′ End DNA Labeling Kit (Thermo Scientific). Northern blot analysis method was described previously (Sambrook et al. 1989). RNA levels were detected using Chemiluminescent Nucleic Acid Detection Module (Thermo Scientific).

### DNA microarrays

To isolate total RNA, the overnight culture of *P. aeruginosa* PA14 wild‐type and the *higA‐*mutant strains were inoculated in 25 mL of LB medium at an initial turbidity of 0.05 at 600 nm. The culture was collected in 2 mL tubes (four tubes) at a turbidity of 2.0 at 600 nm. RNAlater buffer (Applied Biosystems, Foster City, CA) (100 *μ*L) was added immediately into the sample tubes to stabilize RNA, and the sample tubes were rapidly frozen in ethanol/dry ice. Cells were lysed using 0.1 mm zirconia/silica beads and a bead beater (Biospec, Bartlesville, OK) and total RNA was isolated using an RNeasy Mini kit (Qiagen) (Ren et al. 2004). cDNA synthesis, fragmentation, and hybridization to Affymetrix *P. aeruginosa* Genome array (Affymetrix, Santa Clara, CA, USA) were performed as previously described (González Barrios et al. 2006). The gene expression data are accessible through GEO accession number GSE74730.

### Pyocyanin assay

The *P. aeruginosa* PA14 wild‐type, *higA‐*mutant, and negative control (*phzM* and *phzS* mutants) strains were inoculated at a 1/1000 dilution from the overnight culture in LB medium and incubated for 24 h. As described previously (Essar et al. 1990), a 1‐mL sample was centrifuged. The 800 *μ*L of supernatant was extracted with 480 *μ*L of chloroform. The sample was vortexed and centrifuged, then re‐extracted with 0.2 N HCl. The absorbance of this sample was measured at 520 nm. The OD values at 520 nm were normalized with bacteria growth to eliminate any possible growth affects. At least three replicates were performed.

### Pyochelin assays

The *P. aeruginosa* PA14 wild‐type, *higA‐*mutant, and negative control (*pchB* and *pchF* mutants) strains were grown overnight in LB medium. As described previously (Takase et al. 2000), acetic acid (0.3 mL) and dichloromethane (1.5 mL) were added into 3 mL of each culture. The samples were vortexed briefly and centrifuged for 5 min. One milliliter of dichloromethane fractions (bottom layer) were collected and evaporated. The samples were resuspened in 10 *μ*L of dichloromethane and applied onto silica thin‐layer plates for chromatography in chloroform–acetic acid–ethanol (90:5:2.5). The pyochelin spots were scraped from the plates, eluted with 1 mL of methanol, and measured using a spectrophotometer at 313 nm and using a spectrofluorimeter (excitation at 355 nm and emission at 430 nm). At least three replicates were performed.

### Pyoverdine assays

The *P. aeruginosa* PA14 wild‐type, *higA‐*mutant, and negative control (*pvdF* mutant) strains were grown overnight in LB medium. For the chrome azurol S (CAS) agar plate assay (Owen and Ackerley 2011), 1 *μ*L of the overnight culture was placed in the middle of CAS agar plate. After incubation at 37°C for 1 and 2 days, the plate was observed under UV light by the formation of a fluorescent zone around the cells (Yu et al. 2014). For CAS liquid assay (Yu et al. 2014), an overnight culture in LB was diluted to an initial turbidity of 0.1 at 600 nm in King's B medium (Yu et al. 2014). After 1 or 2 days incubation at 37°C, the samples were centrifuged, and the supernatant was used to measure the pyoverdine production using a fluorescence spectrometer (excitation at 405 nm and emission at 460 nm).

### Biofilm assay using crystal violet

Biofilm formation was assayed in 96‐well polystyrene plates using 0.1% crystal violet staining as described previously (Fletcher 1977) with some modifications. Diluted overnight cultures at an initial turbidity of 0.05 at 600 nm were inoculated into 96‐well plates with M9 medium with 0.4% glucose and 0.4% casamino acids (Rodriguez and Tait 1983) and the bacteria were cultured for 48 h without shaking. After the crystal violet was added to each well, the wells were rinsed and dried, and ethanol was added to dissolve the crystal violet. The total biofilm formation samples were measured at 540 nm, whereas cell growth was measured at 620 nm. Biofilm formation was normalized by the bacterial growth to reduce any growth effect. At least two independent cultures were used for each strain.

### Swarming assay

The *P. aeruginosa* PA14 wild‐type, *higA‐*mutant, and negative control (*rhlR* mutant) strains were grown overnight in LB medium. The culture (1 *μ*L) was inoculated in the middle of fresh BM2 plates (Overhage et al. 2008) that were dry for 3 h before inoculation, and the plates were incubated for 18 h. The agar plate coverage was measured using ImageJ software http://http%5c%5c:(www.imagej.nih.gov/ij/). At least three replicates were performed.

## Results

### Identification of the putative HigB/HigA TA system

**Figure 1:**
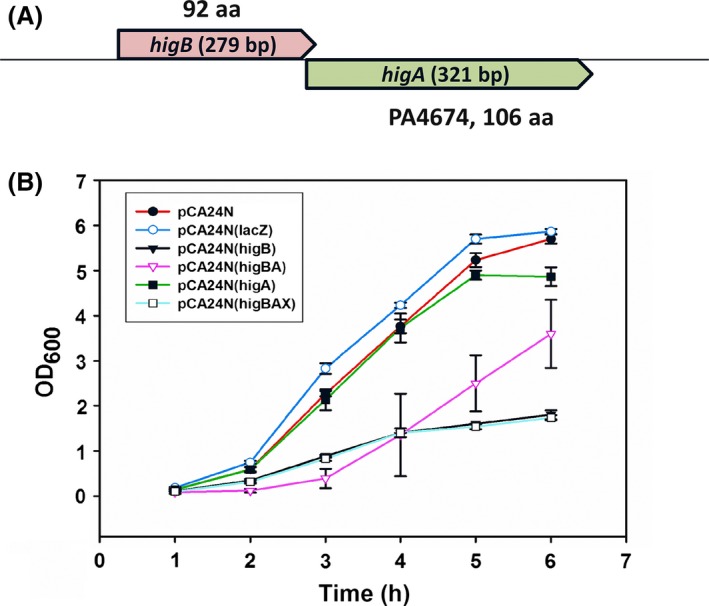
*higBA* loci and results showing HigB toxicity is masked by HigA. (A) The *higBA* operon of *Pseudomonas aeruginosa* PA14. The toxin gene *higB* (not annotated, 5514513‐5514791) and the antitoxin gene *higA* (PA4674, PA14_61840, 5514196‐5514516) were unveiled using RASTA software. (B) Overnight cultures of strains of *Escherichia coli* TG1/pCA24N (pCA24N, control), *E. coli* TG1/pCA24N (lacZ) (pCA24N(lacZ), control), *E. coli* TG1/pCA24N(His‐higB) (pCA24N(higB)), *E. coli* TG1/pCA24N(higA‐FLAG) (pCA24N(higA)), *E. coli* TG1/pCA24N(His‐higB‐higA‐FLAG) (pCA24N(higBA)), and *E. coli* TG1/pCA24N(His‐higB‐higAX‐FLAG), where “X” indicates the translation start signal was changed to a threonine codon so antitoxin HigA is not produced (pCA24N(higBAX)), were inoculated into 25 mL of LB medium at an initial of turbidity of 0.05 at 600 nm at 37°C. IPTG (0.01 mmol/L) was added after 1 h. The error bars shown are standard deviation from three independent cultures.

We identified a putative HigB/HigA system in the genome of *P. aeruginosa* PA14 by using the RASTA Bacteria program (Sevin and Barloy‐Hubler 2007). This program searches for type II TA systems, and the HigB/HigA system got the highest score (90%) which indicates it is likely to be a TA system (scores above 70% are indicated to likely be TA systems). HigB was not annotated previously. HigB is a small protein (92 amino acids), and HigA consists of 106 amino acids (Fig. 1A). Their genes overlap by one nucleotide, which is the same as the HigB/HigA system in *P. vulgaris* (Schureck et al. 2014), and gene overlap is a common characteristic of TA systems (Yamaguchi et al. 2011).

### HigB and HigA form a TA system

To assess whether HigB functions as a toxin and whether HigA masks HigB toxicity, the activity of the two proteins were evaluated in *E. coli*. HigB toxin produced from plasmid pCA24N inhibited the growth in *E. coli* TG1 (Fig. 1B); hence, HigB showed strong toxin activity in this non‐native host. When both HigB and HigA were produced simultaneously, the toxicity of HigB was counteracted (Fig. 1B). Therefore, HigB/HigA is a bona fide TA system.

### Antitoxin HigA binds to toxin HigB and functions as a protein

Antitoxin HigA could mask HigB toxicity as either RNA or as a protein; hence, the start codon of *higA* was converted to Thr and its effect on HigB toxicity was evaluated. Growth in *E. coli* showed that the translation mutation in *higA* abolished its antitoxin activity. Therefore, HigA functions as a protein antitoxin (Fig. 1B).

We then hypothesized that the HigA antitoxin directly binds toxin HigB to inactivate it. To verify the direct interaction between antitoxin HigA and toxin HigB, a pull‐down experiment was performed. The protein HigB from pCA24N(His‐higB) and from pCA24N(His‐higB‐higA‐FLAG) was tagged with six histidines at the N terminus, and the protein HigA from pCA24N(His‐higB‐higA‐FLAG) was tagged using the FLAG octapeptide at the C terminus.

**Figure 2:**
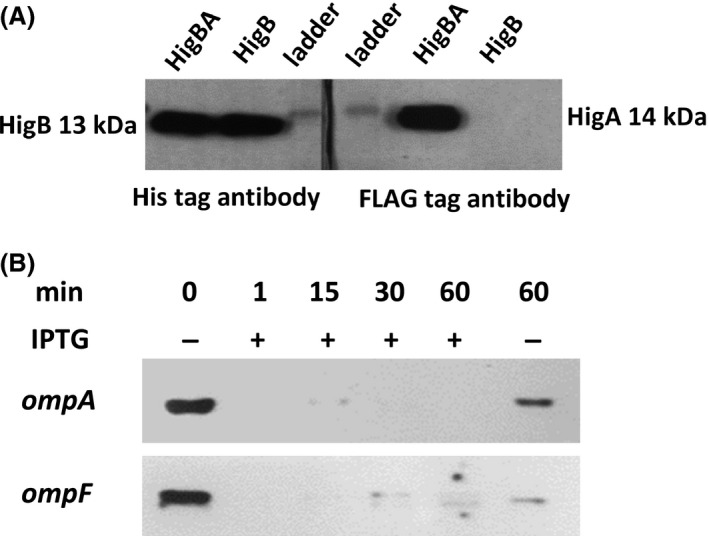
Western blot of HigB/HigA showing HigA binds to HigB and HigB degrades *ompA* and *ompF* mRNA in vivo. *Escherichia coli* TG1/pCA24N(His‐higB) and *E. coli* TG1/pCA24N(His‐higB‐higA‐FLAG) were grown in LB medium at 37°C at an initial turbidity of 0.05 at 600 nm, then 0.1 mmol/L of IPTG was added to produce HigB and HigA for 5 h. (A) The toxin HigB was tagged with six histidines, and the antitoxin HigA was tagged with the FLAG octapeptide. After producing both proteins via the *E. coli* TG1/pCA24N(His‐higB‐higA‐FLAG) strain, HigA binding to HigB was checked based on binding of HigB to the nickel column via its His tag. After purification of HigB, both HigB (left hand panel, using a His antibody) and HigA (right hand panel, using a FLAG antibody) from the HigB/HigA complex (HigBA) were detected independently in a denaturing gel. HigB production from the *E. coli* TG1/pCA24N(His‐higB) strain served as a positive control for HigB detection via the His antibody (left hand panel) as well as a negative control for the absence of HigA detection with the FLAG antibody (right hand panel). (B) RNA was isolated after *higB* was induced with 0.5 mmol/L of IPTG using the *E. coli* TG1/pCA24N(His‐higB) strain for 0, 1, 15, 30, and 60 min. Northern blot analysis was performed for *ompA* and *ompF* RNA detection.

Using the His‐tagged HigB protein to pull down the Flag‐tagged HigA antitoxin, a western blot was performed. Both proteins HigB and HigA were detected using His and FLAG tag antibodies, respectively; hence, antitoxin HigA interacts directly with toxin HigB (Fig. 2A). Therefore, HigB and HigA interact at the protein level and are a type II TA system (i.e., protein–protein TA system).

### Toxin HigB functions as an RNase

To determine the enzyme activity of HigB in *E. coli*, a Northern blot analysis for *ompA* and *ompF* was performed (Fig. 2B); these two loci encode large genes so they are used frequently in TA studies (Hurley and Woychik 2009). The degradation of the mRNAs was detectable immediately after induction (1 min) of *higB*. The level of the control mRNAs at the 60‐min time point without *higB* induction was also decreased due to low‐level activity of the plasmid promoter, but it was not as strong as in the samples with *higB* induction.

### Toxin HigB catalytic sites

**Figure 3:**
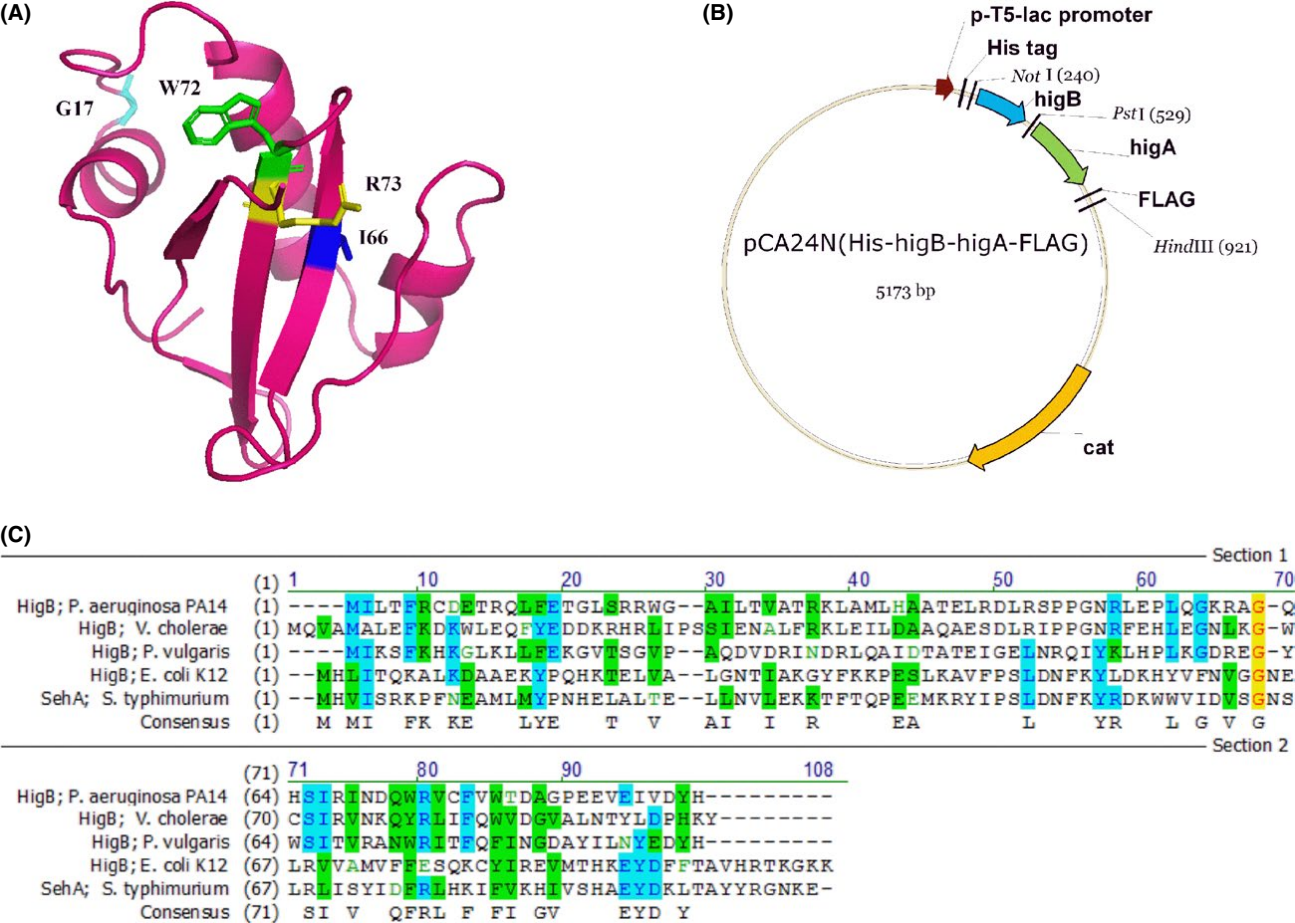
(A) Predictive modeled structure of HigB of *Pseudomonas aeruginosa* PA14 based on HigB structure of *Proteus vulgaris* using Phyre (Moker et al. 2010). The RNase conserved amino acids G17, I66, W72, and R73 that were identified in the mutagenesis experiment are in light blue, blue, green, and yellow, respectively. PyMOL was used to edit the modeled structure. (B) Plasmid map of pCA24N(higBA). The plasmid map showing the pCA24N backbone plasmid containing *higB* and *higA* genes and cloning sites (*Not*I, *Pst*I, and *Hind* III). (C) Alignment of the toxin Hig B protein from *P. aeruginosa* PA14, *Vibrio cholera*,* Proteus vulgaris*, and *Escherichia coli* K12, and SehA from *Salmonella typhimurium*.

Error‐prone PCR of *higB* was performed to generate a library of HigB variants in *E. coli* to determine the residues required for its RNase activity. The HigB variants were selected on LB plates with 0.1 mmol/L of IPTG to produce the toxin; under these conditions, native HigB prevented the formation of colonies. Variants were selected based on their enhanced growth, and the substitutions were identified as G17D/A61E, V28D, R31H, Q57P, Q63H/W72C, H64P, I66N, R67W, W72R, and R73H. The Q63H, H64P, R67W, and W72C substitutions were found multiple times. These results indicate that the amino acids at these positions are crucial for the toxicity of HigB. We also aligned HigB sequences of *P. aeruginosa* PA14, *P. vulgaris*, and *V. cholerae* and found that most of the crucial amino acids we identified in the variants (G17, I66, W72, and R73, Fig. 3A) are conserved or nearby the conserved amino acids which are likely to be the active site of HigB (Schureck et al. 2014).

### Toxin HigB reduces pyochelin production

**Figure 4:**
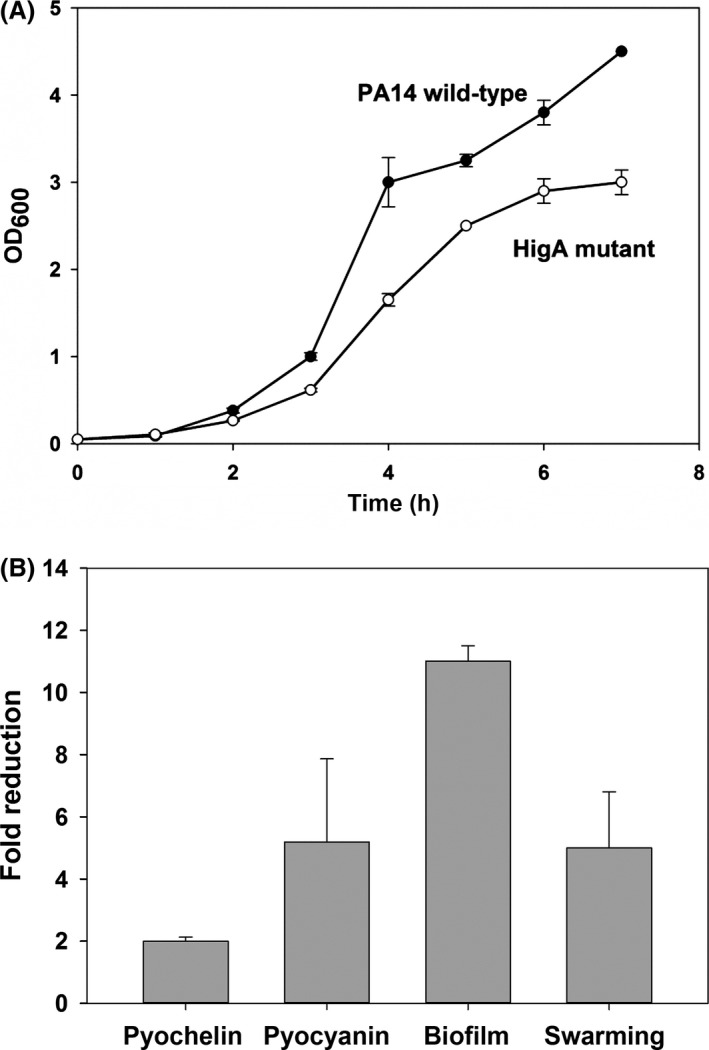
(A) Growth of *Pseudomonas aeruginosa* PA14 and the *higA* mutant. The strains were grown in LB medium at 37°C. At least two replicates were performed. (B) The *higA* mutation reduces pyochelin, pyocyanin, biofilm formation, and swarming. *Pseudomonas aeruginosa* PA14 and the *higA* mutant were grown in LB medium at 37°C. Results from two replicates are shown.

To determine the physiological role of HigB/HigA in *P. aeruginosa*, a whole‐transcriptome experiment was performed for the *higA* antitoxin deletion mutant compared to the *P. aeruginosa* PA14 wild‐type strain. The rationale was that for the strain that lacks the antitoxin, the effect of the toxin could be discerned due to enhanced activity of the toxin. Notably, the specific growth rates of *P. aeruginosa* PA14 and the *higA* antitoxin deletion mutant strain were 1.2 and 0.8 h^−1^, respectively, so there is a modest decrease in growth upon activation of toxin HigB (Fig. 4A).

The microarray results (Table 3) indicate that deletion of the antitoxin induces toxin transcription by 28‐fold, as expected since antitoxins normally repress the TA operon (Brown et al. 2013). Furthermore, the PA2405‐2410 operon, which is part of the pyoverdine locus (Ravel and Cornelis 2003), has the most induced genes upon activating toxin HigB (i.e., deletion of *higA*); these genes probably encode proteins related to membrane transport. However, there was not much difference in pyoverdine production between the wild‐type strain and the *higA* mutant (data not shown) which collaborated previous work which reported that the mutations in this PvdS‐regulated PA2403‐PA2410 cluster did not affect pyoverdine production (Ravel and Cornelis 2003).

**Table 3:** Summary of the largest fold changes in gene expression for the *higA* mutant versus the isogenic *Pseudomonas aeruginosa* PA14 wild type strain. Both strains were grown to a turbidity of 2.0 at 600 nm in LB medium

Genes	Fold change	Description
*higB* 28.0 toxin gene		
Part of the pyoverdine locus (Ravel and Cornelis 2003)		
PA2405	9.2	Hypothetical protein
PA2404	8.0	Hypothetical protein; membrane proteins
PA2408	7.0	Probable ATP‐binding component of ABC transporter, membrane proteins
PA2403	5.7	Hypothetical protein; membrane proteins
PA2406	5.3	Hypothetical protein
PA2409	4.0	Probable permease of ABC transporter, membrane proteins/transport of small molecules
PA2407	3.5	Probable adhesion protein, motility and attachment
PA2410	3.5	Hypothetical protein
Related to pyoverdine and iron transportation		
PA2398_*fpvA*	7.0	Ferripyoverdine receptor/transport of small molecules
PA4675	5.3	Probable TonB‐dependent receptor/transport of small molecules
PA0805	4.0	Hypothetical protein
Pyochelin genes		
PA4230_*pchB*	−9.2	Salicylate biosynthesis protein PchB/transport of small molecules; secreted factors (toxins, enzymes, alginate)
PA4231_*pchA*	−7.5	Salicylate biosynthesis isochorismate synthase/secreted factors (toxins, enzymes, alginate); transport of small molecules
PA4229_*pchC*	−6.1	Pyochelin biosynthetic protein PchC/transport of small molecules; secreted factors (toxins, enzymes, alginate)
PA4226_*pchE*	−4.9	Dihydroaeruginoic acid synthetase/transport of small molecules; secreted factors (toxins, enzymes, alginate)
PA4224_*pchG*	−4.9	Hypothetical protein; membrane proteins
PA4228_*pchD*	−4.6	Pyochelin biosynthesis protein PchD/transport of small molecules; secreted factors (toxins, enzymes, alginate)
PA4225_*pchF*	−4.0	Pyochelin synthetase/transport of small molecules; secreted factors (toxins, enzymes, alginate)
PA4227_*pchR*	−2.6	Transcriptional regulator PchR/transcriptional regulators
Pyochelin‐related genes and nearby genes to the *pch* operon		
PA4221_*fptA*	−6.1	Fe(III)‐pyochelin receptor precursor/transport of small molecules
PA4223	−4.0	Probable ATP‐binding component of ABC transporter/membrane proteins; transport of small molecules
PA4218	−4.0	Probable transporter/membrane proteins; transport of small molecules
PA4220	−3.7	Hypothetical protein
PA4219	−3.5	Hypothetical protein/membrane proteins
PA4222	−3.2	Probable ATP‐binding component of ABC transporter/transport of small molecules
PA0985_*pyoS5*	−3.2	Probable colicin‐like toxin/membrane proteins; secreted factors (toxins, enzymes, alginate)

Critically, the microarray data show that pyochelin‐related genes (*pchA*,* pchB*,* pchC*,* pchD*,* pchE*,* pchF*,* pchG*,* pchR*, and *fptA*), which encode proteins related to iron transfer, are repressed upon activating the toxin HigB. Pyochelin is a siderophore produced by *P. aeruginosa* which increases the growth and lethality of pathogenic bacteria (Cox 1982). FptA is the receptor for ferric pyochelin, and its expression is pyochelin dependent (Heinrichs and Poole 1996). A number of studies have shown that there is a correlation between pyochelin and virulence (Cox 1982; Sokol 1987; Wang et al. 1996). Pyochelin levels were assayed in the *higA* mutant and found to be reduced by 2.0 ± 0.1‐fold (Fig. 4B); hence, the microarray data were corroborated by the reduced pyochelin levels. The OD_313_ values for the pyochelin assay for *P. aeruginosa* PA14 and the *higA* mutant were 0.104 and 0.05, respectively. Therefore, the HigB toxin reduces virulence via a reduction in pyochelin, and HigA antitoxin represses its own transcription. These results were not affected by growth since the samples for pyochelin assay were collected in the stationary phase at the same turbidity.

### Toxin HigB reduces pyocyanin production

Pyocyanin is a blue redox‐active secondary metabolite that interferes with multiple cellular functions and has crucial roles in *P. aeruginosa* infections (Lau et al. 2004). The *higA* antitoxin deletion mutant was less blue compared to the wild type; hence, chloroform extraction was performed to more accurately measure the amount of the pyocyanin in an overnight culture. The *higA*‐mutant strain produced 5 ± 3‐fold less pyocyanin than the *P. aeruginosa* PA14 wild‐type strain (Fig. 4B), which showed that the toxin HigB affected the pyocyanin level in *P. aeruginosa*. The OD_520_/OD_600_ values for the pyocyanin assay for *P. aeruginosa* PA14 and the *higA* mutant were 0.0086 ± 0.0012 and 0.0018 ± 0.00007, respectively. These results were not affected by growth because the samples were collected after 24 h at the same turbidity.

### Toxin HigB reduces biofilm formation and swarming motility

**Figure 5:**
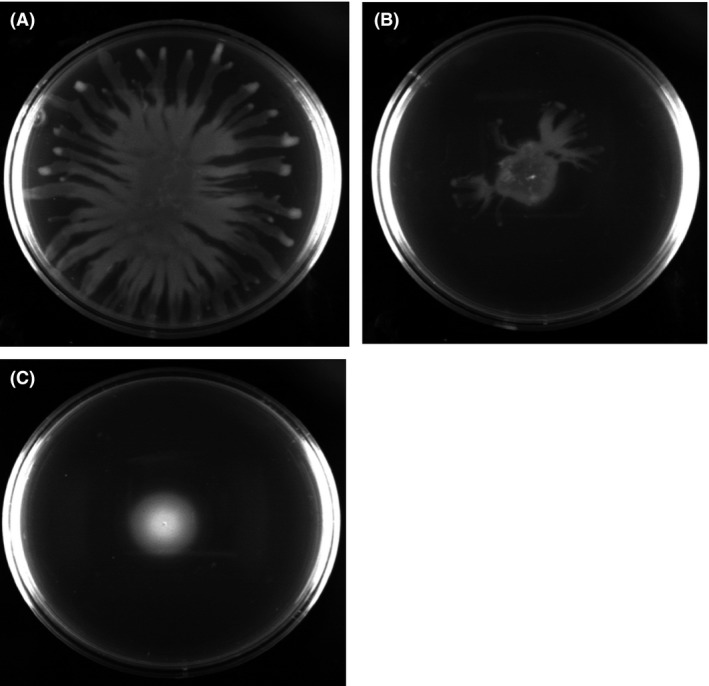
The *higA* mutant has less swarming compared to the wild‐type strain. *Pseudomonas aeruginosa* PA14, the *higA* mutant, and the negative control (*rhlR* mutant) were grown overnight in LB medium. The culture (1 *μ*L) was inoculated into the middle of a BM2 plate (Overhage et al. 2008) that was dried for 3 h before inoculation, and the plates were incubated for 18 h. Swarming plates of (A) *P. aeruginosa* PA14 with 52 ± 8% area coverage, (B) the *higA* mutant with 11 ± 2% area coverage, and (C) the *rlhR* mutant (negative control) with 5 ± 0.4% area coverage. Results shown are from one of three representative independent cultures.

Since TA systems are related to biofilm formation (Ren et al. 2004; González Barrios et al. 2006; Kim et al. 2009) as well as quorum sensing and motility (González Barrios et al. 2006), biofilm formation and swarming were investigated for the HigB/HigA TA system. After 48 h in M9 medium with 0.4% glucose and 0.4% casamino acids (Rodriguez and Tait 1983), the *higA* mutant had 11.0 ± 0.5‐fold less normalized biofilm than the *P. aeruginosa* PA14 wild‐type strain (Fig. 4B). The OD_540_/OD_600_ values for the biofilm assay for *P. aeruginosa* PA14 and the *higA* mutant were 1.3 ± 0.4 and 0.12 ± 0.04, respectively. These results were not affected by growth because the samples were collected after 48 h, and the planktonic cell densities were nearly identical. Furthermore, the *higA* mutant had 5 ± 2‐fold lower swarming motility than the *P. aeruginosa* PA14 wild‐type strain (Figs. 4B and 5). Hence, toxin HigB reduces both biofilm formation and swarming.

## Discussion

We present additional evidence in this report that TA systems are involved in pathogenicity. Furthermore, we find that the HigB/HigA TA system of *P. aeruginosa* affects its virulence in a manner that is distinct from the way any other TA system has been linked to virulence, since here we found HigB/HigA affects virulence through pyochelin, pyocyanin, swarming (as well as through biofilm formation). We also demonstrate clearly that the *P. aeruginosa* HigB/HigA system is a type II bona fide TA system.

*Pseudomonas aeruginosa* produces two siderophores, pyoverdine and pyochelin (Ankenbauer and Quan 1994), and gene expression related to both siderophores was affected by the HigB/HigA TA system. Based on our microarray results, *fpvA*, which encodes the receptor of the siderophore ferripyoverdine, was induced in the *higA* mutant compared to the *P. aeruginosa* PA14 wild‐type strain; this would make cells with the HigB toxin activated more susceptible to pyocins S2 and S3 since these pyocins use the ferripyoverdine receptor (Denayer et al. 2007). In contrast, *fptA*, which encodes the receptor of the siderophore pyochelin, was reduced in the *higA* mutant. *pyoS5*, which encodes a toxin that uses the FptA ferripyochelin receptor to enter the cell (Elfarash et al. 2014), was also repressed.

The HigB toxin is prevalent in pseudomonads; for example, *P. aeruginosa* DK2, *P. aeruginosa* B136‐33, *Pseudomonas stutzeri* DSM 10701, *Pseudomonas putida* F1, *Pseudomonas putida* KT2440, *Pseudomonas fluorescens* F113, and *Pseudomonas denitrificans* all contain genes for the toxin. Although the genes for putative HigB/HigA TA systems have been found in many organisms, most of them have not been characterized. An exception is the HigB toxin from *Proteus vulgaris* which has a highly conserved residue N71 for controlling mRNA specificity by interacting with the 16S rRNA residue C1054 (Schureck et al. 2015). However, the HigB from *P. aeruginosa* has glutamine at this position. The HigB toxin protein we found in *P. aeruginosa* PA14 was aligned with the HigB from *V. cholera*,* Proteus vulgaris*,* E. coli* K12, and SehA from *S. typhimurium* to reveal that there is only 34.3%, 28%, 10.6%, and 8.7% identity, respectively (Fig. 3C). Hence, the *P. aeruginosa* system that we describe here is distinct since proteins that share about 20% identity are not related, in that only those with identities of 50% or greater are usually considered related in databases (Seffernick et al. 2001). Therefore, we have characterized a novel HigB/HigA TA family member and shown how the TA system affects virulence factors of an important opportunistic pathogen. This study also represents the first TA system that has been characterized in *P. aeruginosa*.

## Conflict of Interest

None declared.
